# Impact of clinical input variable uncertainties on ten-year atherosclerotic cardiovascular disease risk using new pooled cohort equations

**DOI:** 10.1186/s12872-016-0352-x

**Published:** 2016-08-31

**Authors:** Himanshu Gupta, Chun G. Schiros, Oleg F. Sharifov, Apurva Jain, Thomas S. Denney

**Affiliations:** 1 Department of Medicine, Cardiovascular Disease, University of Alabama at Birmingham, 1808 7th Ave South, BDB 101, Birmingham, AL 35294 USA; 2 VA Medical Center, Birmingham, AL USA; 3 School of Public Health, The University Of Texas Health Science Centre, Houston, TX USA; 4 Department of Electrical and Computer Engineering, Auburn University, Auburn, AL USA

**Keywords:** Cholesterol, Statins, Cardiovascular disease, Atherosclerosis, Primary prevention, Computer simulations

## Abstract

**Background:**

Recently released American College of Cardiology/American Heart Association (ACC/AHA) guideline recommends the Pooled Cohort equations for evaluating atherosclerotic cardiovascular risk of individuals. The impact of the clinical input variable uncertainties on the estimates of ten-year cardiovascular risk based on ACC/AHA guidelines is not known.

**Methods:**

Using a publicly available the National Health and Nutrition Examination Survey dataset (2005–2010), we computed maximum and minimum ten-year cardiovascular risks by assuming clinically relevant variations/uncertainties in input of age (0–1 year) and ±10 % variation in total-cholesterol, high density lipoprotein- cholesterol, and systolic blood pressure and by assuming uniform distribution of the variance of each variable. We analyzed the changes in risk category compared to the actual inputs at 5 % and 7.5 % risk limits as these limits define the thresholds for consideration of drug therapy in the new guidelines. The new-pooled cohort equations for risk estimation were implemented in a custom software package.

**Results:**

Based on our input variances, changes in risk category were possible in up to 24 % of the population cohort at both 5 % and 7.5 % risk boundary limits. This trend was consistently noted across all subgroups except in African American males where most of the cohort had ≥7.5 % baseline risk regardless of the variation in the variables.

**Conclusions:**

The uncertainties in the input variables can alter the risk categorization. The impact of these variances on the ten-year risk needs to be incorporated into the patient/clinician discussion and clinical decision making. Incorporating good clinical practices for the measurement of critical clinical variables and robust standardization of laboratory parameters to more stringent reference standards is extremely important for successful implementation of the new guidelines. Furthermore, ability to customize the risk calculator inputs to better represent unique clinical circumstances specific to individual needs would be highly desirable in the future versions of the risk calculator.

**Electronic supplementary material:**

The online version of this article (doi:10.1186/s12872-016-0352-x) contains supplementary material, which is available to authorized users.

## Background

The recent American College of Cardiology/American Heart Association (ACC/AHA) guideline on the treatment of blood cholesterol to reduce atherosclerotic cardiovascular disease (ASCVD) risk in adults recommends the use of the new pooled cohort equations to calculate ten-year risk to help define the population cohorts that are likely to benefit from either the initiation of statin therapy in non-diabetics or define the intensity of statin therapy in patients with diabetes for the primary prevention of ASCVD [1, 2]. These equations were derived from analyzing five major longitudinal studies that include the Framingham Heart Study (FHS and offspring cohort) [3–5], the Coronary Artery Risk Development in Young Adults (CARDIA) [6], the Cardiovascular Health Study (CHS) [7], and the Atherosclerosis Risk in Communities Study (ARIC) [8]. The equations incorporate sex-and race-specific proportional hazards models consisting of covariates of objectively measured values of systolic blood pressure (BP), total-cholesterol (c) and HDL-c with other clinical and demographic features to calculate ten-year risk of ASCVD. A risk calculator is available for download [http://my.americanheart.org/cvriskcalculator].

The ten- year risk assessment has profound implications for clinical decision-making for an individual patient and for formulating health policies for primary prevention [9, 10]. Application of the pooled cohort equations to the National Health and Nutrition Examination Survey (NHANES) dataset from 2007 to 2010 reveals that approximately 20 % of the US population (about 20 million people) have predicted ten- year risk between 5 and 9.9 % and are therefore potential candidates for statin therapy [11]. Despite multiple recent analyses that suggest good calibration in general population based cohorts [12–14], there is a considerable ongoing debate about the value of the new pooled cohort equations as a tool to define thresholds for drug therapy including the major impact of advanced age on calculated risk [15]. When the risk equations are applied to a distinct population cohort different from original studied cohorts, there has been conflicting data. Application of these risk equations to the Reasons for Geographic and Racial Differences in Stroke (REGARDS) cohort demonstrated that observed and predicted CVD risks at 5 years were similar suggesting that these equations are well calibrated with moderate to good discrimination [14]. In contrast when the risk equations are applied to the Multi-Ethnic Study of Atherosclerosis (MESA) cohort, there appears to be an overestimation of risk and a lack of superior calibration or discrimination compared with the older risk scores [16]. We have recently published in-depth analysis of the ten-year risk equations [17] and also described a modified treatment approach based on ten year risk assessment [18].

Because risk equations represent mathematical best fit based on the results of prospective cohort studies, certain inherent uncertainties (i.e., predictive intervals) always exist when applying group equation to the individual. This aspect has been highlighted in the ten-year risk guidelines and discussed elsewhere [1, 2, 19]. Another important aspect of the new pooled cohort equations that has not been well described is the influence of the uncertainties in clinical input measurements of the discrete variables that are needed for risk calculation on ten-year risk. Age in the longitudinal studies is usually expressed in years corresponding to the last birthday which would indicate that there can be a variance of up to 1 year compared to actual age (for example 60.75 years = 60 years, indicating difference of 0.75 years). BP measurement is prone to a number of errors and uncertainties [15]. Furthermore, in CARDIA, ARIC and CHS, a random zero sphygmomanometer was used that produces readings 2–3 mmHg lower than manual sphygmomanometer [20, 21]. In contrast, in FHS, BP measurements were made with a mercury-column sphygmomanometer and the average of two physician-obtained measures constituted the examination BP. This approach is markedly different from routine clinical practice. Similarly for total-c and HDL-c, the measurement results in longitudinal studies were generally standardized to those of a reference laboratory. The National Cholesterol Education Program (NCEP) guidelines recommend total analytical error in clinical models for the measurement of total-c of ≤ 9.6 % and HDL-c of ≤ 13.3 % [22]. These operating characteristics may not hold true for many commercial assays [23]. Moreover the clinical labs are certified to Clinical Laboratory Improvement Amendment (CLIA) standards where the acceptable total error for total-c is ±10 %, and for HDL-c is ±25 % [24].

Based on the hazard ratio of each variable to the ten-year risk [1, 2], the variations/uncertainties in age, systolic BP, HDL-c and total-c may have a significant influence on the ten-year ASCVD risk. It is therefore conceivable that due to the uncertainties in the input values of these variables in routine clinical practice, there is variable categorization of individuals into a high or low risk grouping, which in turn may cause erroneous management decisions based on the guidelines. Therefore it is important to define the effects of the input uncertainties to the risk calculation. Here, we evaluate the influence of these uncertainties on the ten-year risk and hence on the proposed treatment algorithms.

## Methods

### Study dataset

We used the publicly available NHANES dataset (2005–2010). Participants with all the variable values required for ten- year risk calculation between ages 40–75 years were included (*n* = 2355, Table 1 describes the baseline characteristics). Age was reported based on last birthday (i.e., age in completed years) calculated by subtracting the date of birth from the reference date, with the reference date being the date of contact with an individual. Gender and treatment for hypertension was self-reported. Diabetes mellitus (DM) included self-reported physician diagnosis or fasting plasma glucose of ≥126 mg/dL or a hemoglobin A1c ≥ 6.5 %. Current smokers were persons who smoked 100 cigarettes and who currently smoked every day or some days. Race was self-reported based on 1997 Revisions to the Standards for the Classification of Federal Data on Race and Ethnicity [25]. Total-c and HDL-c measurements were using standard methods as described [26]. Individuals with self-reported coronary artery disease, heart attack (or myocardial infarction), angina and stroke were excluded.

**Table 1:** Baseline characteristics

Variable	Entire cohort (*N* = 2355)	Without Hispanic Ethnicity (*N* = 1805)
AA/White/Hispanic, %	29/48/23	38/62/0
Male/Female, %	45/55	46/54
Age, yrs	60 ± 10	60 ± 10
Total Cholesterol, mg/dl	200 ± 41	199 ± 41
HDL Cholesterol, mg/dl	53 ± 17	54 ± 17
Blood Pressure, mmHg	133 ± 20	133 ± 20
Diabetes, %	32	28
Smoker, %	16	17
Hypertension, %	89	90

Values are n, % or mean ± standard deviation

*AA* African-American

### Pooled cohort equations analysis

**Fig. 1:**
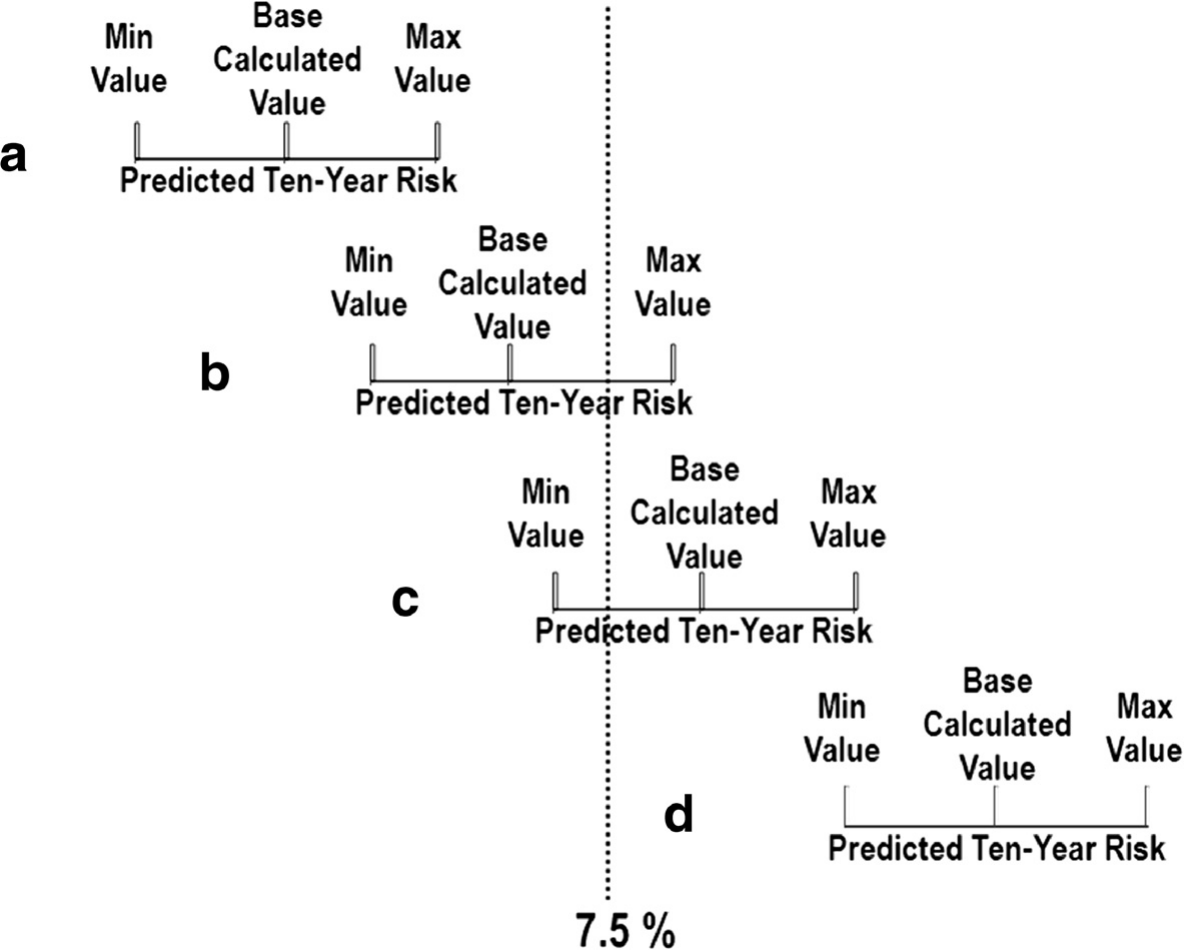
Illustration of four classification scenarios according to boundary limit of 7.5 % due to the uncertainty of clinical measurements on predicted ten-year atherosclerotic cardiovascular disease risk using the new pooled cohort equations. Scenario **a**, the base calculated ten-year risk is well below the boundary limit, the variation in clinical measurements does not result in the change in risk category; For scenario **b** when the base calculated ten-year risk is below or close to the boundary limit, and scenario **c** when the base calculated ten-year risk is equal to or slightly beyond the boundary limit, the variation in clinical measurements may result in the change in risk category; Scenario **d**, the base calculated ten-year risk is well beyond the boundary limit, the variation in clinical measurements does not result in the change in risk category

The new pooled cohort equations were implemented in a custom software package (MATLAB, Natick, MA). Our version of the risk calculator is available online [18]. Predicted ten-year risk for a given set of parameters for the NHANES database (called ‘base calculated’ risk in this paper) along with possible maximum and minimum risks were computed by assuming a variation in age of 0–1 year, and a ± 10 % variation in total-c, HDL-c, and systolic BP. The change in risk category at 5 % and/or 7.5 % risk boundary limits were analyzed. These boundary limits were chosen as these define thresholds for discussion of drug therapies in the new guidelines. For the patient cohort with base calculated risk < (less than) the boundary limits, the percentage of the designated patient cohort that had maximum possible risk ≥ (greater or equal to) the boundary limits indicated the potentially re-categorized population that may be eligible for more intensive therapy but were deemed lower risk based on base calculated measurement (Fig. 1). On the other hand, for the patient cohort with base calculated risk ≥ the boundary limits, the percentage of the designated population that had minimum possible risk < the boundary limits indicated the re-categorized population that may be eligible for more conservative therapy but were deemed higher risk based on baseline calculated measurement (Fig. 1). For the primary analysis, we analyzed white and African American (AA) ethnicity (combined whites and AA *N =* 1805) because the ACC/AHA risk guidelines were primarily based on the white/AA population. We also performed secondary analysis for all participants that includes Hispanic ethnicity (*n* = 2355) (Data Supplement).

### Statistical analysis

The change in risk category at 5 % and/or 7.5 % risk boundary limits were assessed by the Fisher’s exact test (SAS 9.4). A *P* < 0.05 was considered statistically significant. Total number of cohort with possible risk category changes was defined as the total number of differences in base calculated risk and maximum risk in the patient cohort with base calculated ten-year risk < the boundary limits (i.e., for 7.5 % boundary limit, N_[Base Calculated Risk <7.5%]_ –N_[no change in risk categorization compared to base risk <7.5%]_) and differences in base calculated risk and minimum risk in the patient cohort with baseline calculated ten-year risk ≥ the boundary limits (i.e., for 7.5 % boundary limit, N_[Base Calculated Risk ≥7.5%]_ –N_[no change in risk categorization compared to base risk ≥7.5%]_). Percentage of total risk categorization changes was defined as the percent total number of cohort with possible risk category changes to the total patient cohort.

## Results

**Fig. 2:**
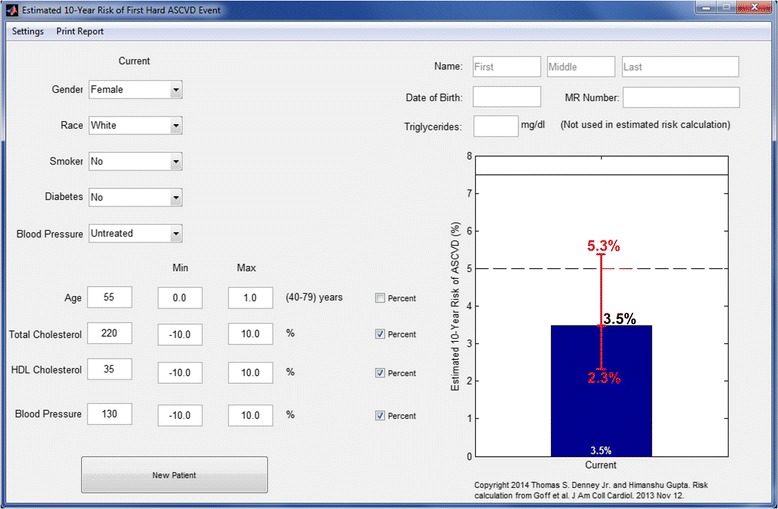
Example illustrating the modified calculator with customizable uncertainty limits for a white female with baseline calculated ten-year risk of 3.5 %. The uncertainty in the measurement values of age, total-c, HDL-c and BP can be input using this customizable tool. The blue bar depicts the calculated baseline ten-year risk, and the red bar represents the maximum and minimum risk. In the depicted example, maximum and minimum risks were computed by assuming variations in input of age (0–1 year) and ± 10 % variation in total-cholesterol (c), HDL-c, and systolic blood pressure. Boundary limits of 5 % and 7.5 % are marked by dashed and solid line, respectively. Our version of the risk calculator is available online [18]

**Fig. 3:**
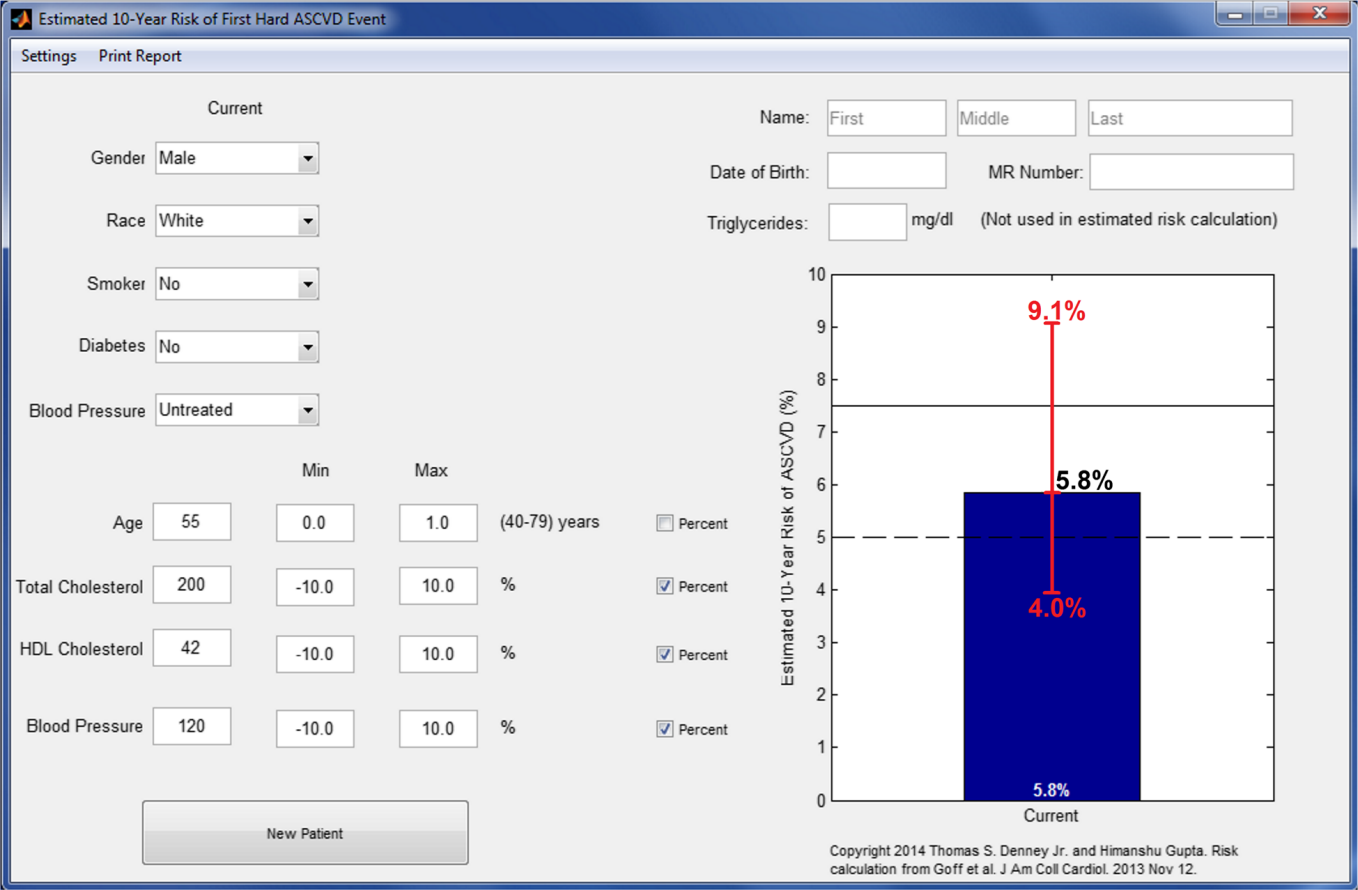
Example illustrating the modified calculator with customizable uncertainty limits for a white male with baseline calculated ten-year risk of 5.8 %. Explanations and abbreviations as in Fig. 2

**Fig. 4:**
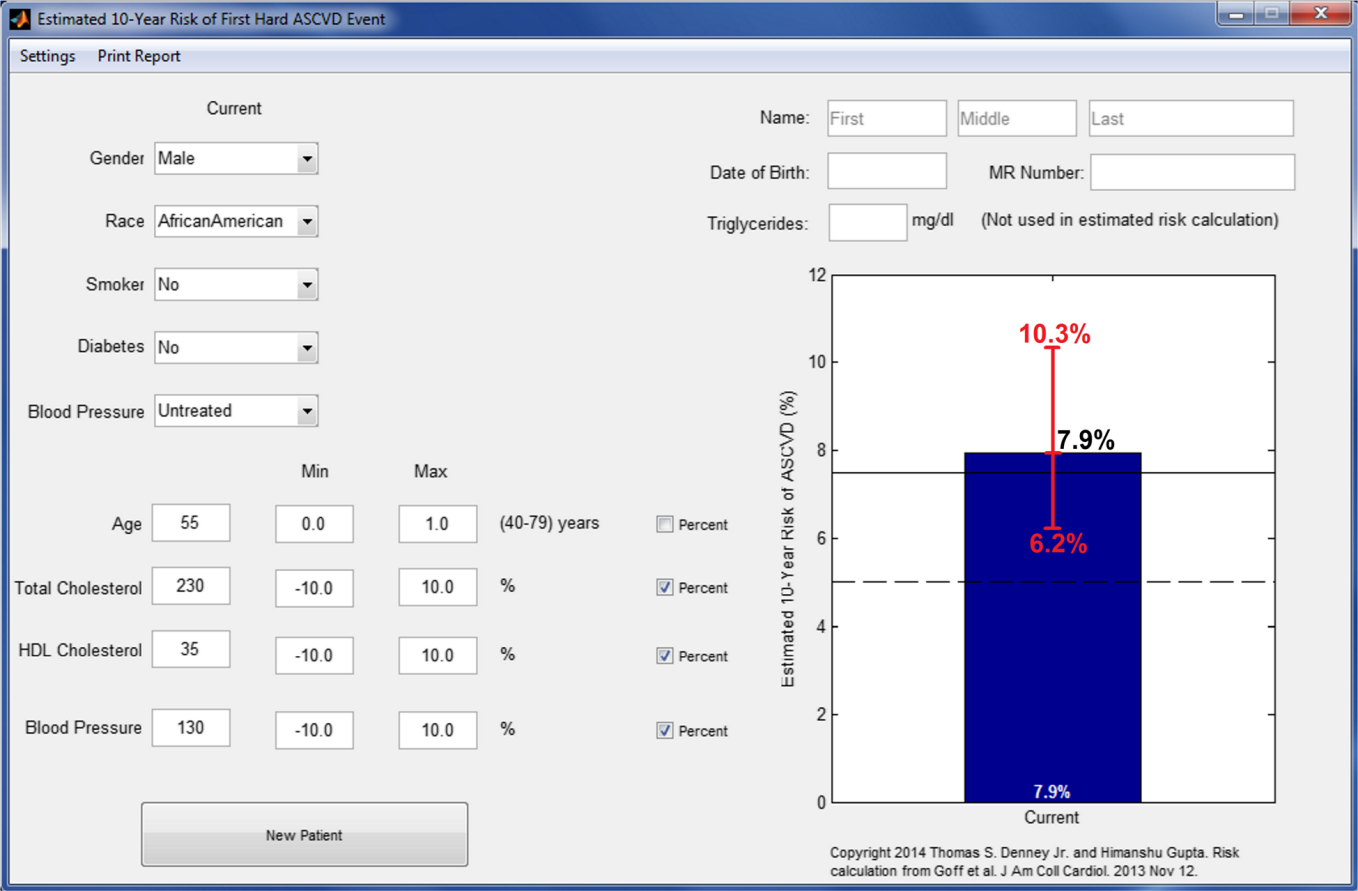
Example illustrating the modified calculator with customizable uncertainty limits for African American male with baseline calculated ten-year risk of 7.9 %. Explanations and abbreviations as in Fig. 2

In Figs. 2, 3 and 4, we provide the examples of application of the modified calculator [18] with customizable uncertainty limits for the realistic case scenarios. For these case scenarios, the calculated maximum and minimum risk based on the variations/ uncertainties of rounding of age and measurements of total-c, HDL-c and systolic BP reveals that the upper and lower boundary limits of ten-year risk crosses the 5 % and 7.5 % boundary limits (Figs. 2, 3 and 4). Thus, due to effect of input variable uncertainties, base-calculated risk category could be potentially increased from <5 % to ≥5 % for patient in Fig. 2 or decreased from ≥7.5 % to <7.5 % in patient in Fig. 4. For example depicted in Fig. 3 with base-calculated risk of 5.8 %, risk category could potentially range from low risk (<5 % boundary limit) to a higher risk (≥7.5 % boundary limit).

Using modified calculator [18], we determined the base and the upper and lower boundary limits of ten-year risk for our study cohort. Baseline characteristics of the participant cohort are described in Table 1. Our detailed analysis dataset of NHANES data is attached as Additional file 1.

For the primary analysis, we analyzed white and AA ethnicity (combined *N =* 1805). We find that around 33 % of the total cohort had base calculated risk of < 7.5 % while the other 67 % had base calculated risk ≥7.5 %. On evaluating the possible risk category changes, up to 38 % of the cohort with base calculated risk <7.5 % (12.57 % of total cohort) may have ≥7.5 % risk based on possible risk. These may therefore need to be treated more aggressively. Furthermore, up to 17 % of the cohort with base calculated risk ≥7.5 % (12.36 % of total cohort) may be re-categorized based on their possible minimum risk, indicating that these individuals may not be treated appropriately. This trend was consistently noted across all subgroups except for African American males where most of the cohort had ≥ 7.5 % baseline risk regardless of the variation in the variables (Table 2).

**Table 2:** Analysis of the impact of input variable variations in categorizing subjects based on ten-year risk threshold of 7.5 %

Patient groups	Base Calculated Ten Year Risk < 7.5 % (% of total)			Base Calculated Ten Year Risk ≥ 7.5 % (% of total)			Total Change of Risk Categorization (% of total)
	Base Calculated Risk <7.5 %	No change of risk categorization (Maximal calculated risk <7.5 %)	Change of risk categorization (Maximal calculated risk ≥7.5 %)	Base Calculated Risk ≥7.5 %	No change of risk categorization (Minimal calculated risk ≥7.5 %)	Change of risk categorization (Minimal calculated Risk <7.5 %	
All (*n* = 1805)	32.96	20.39***	12.57	67.04	55.68***	11.36	23.93
Non-DM (*n* = 1292)	41.80	26.55***	15.25	58.20	46.90***	11.3	26.55
AA (*n* = 426)	36.15	20.42***	15.73	63.85	52.11***	11.74	27.46
AA Male (*n* = 196)	14.29	6.63*	7.66	85.71	78.57	7.14	14.80
AA Female (*n* = 230)	54.78	32.17***	22.61	45.22	29.57***	15.65	38.26
White (*n* = 866)	44.57	29.56***	15.01	55.43	44.34***	11.09	26.10
White Male (*n* = 404)	34.90	19.31***	15.59	65.10	52.72***	12.38	27.97
White Female (*n* = 462)	53.03	38.53***	14.5	46.97	37.01***	9.96	24.46
DM (*n* = 513)	10.72	4.87***	5.85	89.28	77.78***	11.5	17.35
AA (*n* = 255)	5.88	2.35	3.53	94.12	84.71***	9.41	12.94
AA Male (*n* = 107)	0.00	0.00	0	100.00	98.13	1.87	1.87
AA Female (*n* = 148)	10.14	4.05	6.09	89.86	75.00**	14.86	20.95
White (*n* = 258)	15.50	7.36***	8.14	84.50	70.93***	13.57	21.71
White Male (*n* = 130)	10.77	3.85	6.92	89.23	81.54	7.69	14.62
White Female (*n* = 128)	20.31	10.94	9.37	79.69	60.16**	19.53	28.91

Values are % or n. Base calculated: predicted ten-year risk using the raw NHANES data; Minimal Risk: minimum predicted ten-year risk computed by the calculator assuming a variation in age of 0–1 year, and ± 10 % variation in total-cholesterol (c), HDL-c, and systolic blood pressure (BP); Maximal Risk: maximum predicted ten-year risk computed by the calculator assuming a variation in age of 0–1 year, and ± 10 % variation in total-cholesterol (c), HDL-c, and systolic blood pressure (BP); DM: Diabetes mellitus; AA: African-American; Comparisons between Base versus Max/Min Risk were performed using Fisher’s Exact Test; * for *P* < 0.05, ** for *P* < 0.01, and *** for *P* < 0.001

We also calculated possible changes in risk category based on the variation described at 5 % boundary limit for non-diabetics (Table 3).

**Table 3:** Analysis of the impact of input variable variations in categorizing subjects based on ten-year risk threshold of 5 %

Patient groups	Base Calculated Ten Year Risk < 5 % (% of total)			Base Calculated Ten Year Risk ≥ 5 % (% of total)			Total Change of Risk Categorization (% of total)
	Base Calculated Risk <5 %	No change of risk categorization (Maximal calculated risk <5 %)	Change of risk categorization (Maximal calculated risk ≥5 %)	Base Calculated Risk ≥5 %	No change of risk categorization (Minimal calculated risk ≥5 %)	Change of risk categorization (Minimal calculated Risk <5 %	
Non-DM (*n* = 1292)	28.79	16.02***	12.77	71.21	59.83***	11.38	24.15
AA (*n* = 426)	23.47	10.80***	12.67	76.53	65.49***	11.04	23.71
AA Male (*n* = 196)	4.08	1.53	2.55	95.92	90.82	5.1	7.65
AA Female (*n* = 230)	40.00	18.70***	21.3	60.00	43.91***	16.09	37.39
White (*n* = 866)	31.41	18.59***	12.82	68.59	57.04***	11.55	24.36
White Male (*n* = 404)	22.77	10.89***	11.88	77.23	66.34***	10.89	22.77
White Female (*n =* 462)	38.96	25.32***	13.64	61.04	48.92***	12.12	25.76

Values are % or n. Base calculated: predicted ten-year risk using the raw NHANES data; Minimal Risk: minimum predicted ten-year risk computed by the calculator assuming a variation in age of 0–1 year, and ± 10 % variation in total-cholesterol (c), HDL-c, and systolic blood pressure (BP); Maximal Risk: maximum predicted ten-year risk computed by the calculator assuming a variation in age of 0–1 year, and ± 10 % variation in total-cholesterol (c), HDL-c, and systolic blood pressure (BP); DM: Diabetes mellitus; AA: African-American; Comparisons between Base versus Max/Min Risk were performed using Fisher’s Exact Test; * for *P* < 0.05, ** for *P* < 0.01, and *** for *P* < 0.001

We find similar trends and results for possible risk re-categorization as for 7.5 % boundary limit for the total cohort and the subgroups except for African American males for <5 % risk where the number of possible change in risk category did not reach statistical significance as most of the cohort had ≥ 5 % baseline risk regardless of the variation in the variables. When we incorporated Hispanics and calculated the risk based on white cohort (as per the guideline recommendations), we find that the results remained consistent (Additional file 2: Table S1 and Additional file 3: Table S2).

## Discussion

Our analysis of the new-pooled cohort equations for ten-year ASCVD risk quantification provides important caveats that need to be considered: a) The variations/ uncertainties in the input values of continuous variables (age, systolic BP, total-c and HDL-c) used for ten-year risk calculation have an important effect on the calculated ten-year risk; b) At the proposed important decision nodes of 5 % and 7.5 % ten-year risk, we find that these variations/ uncertainties in the input values can influence the categorization into a high or low risk grouping in a substantial number of people. This therefore may have important effects on treatment planning and preventive policies. Uncertainty is a quantification of the doubt about the measurement results [27]. Any parameter which influences the risk calculation, and whose value we do not know precisely, is a source of uncertainty [1]. We report our results based on 0–1 year uncertainty, assuming that the age of individual is typically rounded or truncated based on how his date of birth compared to the date of encounter. The ACC/AHA risk calculator allows for inputting precise age in years (using decimal numbers) which is likely to reduce the uncertainty in the absolute calculated risk and should therefore be taken into account when calculating ten-year risk. Defining uncertainties in routine clinical practice of systolic BP, total-c and HDL-c for calculating 10-year risk is more challenging. The measurements uncertainty (coefficient of variance) of a particular assay is generally reported by the manufactures and similar test characteristics should therefore be replicated wherever the test is performed in a clinical setting. In clinical reports these known uncertainty/variability due to known test characteristics are not generally reported. There are additional parameters that can affect the certainty of the measurements, which may be due to biological factors and/environmental factors or other undefined reasons, and are therefore difficult to quantify. This latter aspect is likely more important for BP measurements. Guidelines have been proposed regarding optimal techniques for BP measurement including instrumentation [15]. Adhering to these guidelines may reduce some of the well described uncertainties in the clinical BP measurements [15, 28]. Although CLIA standards are frequently used for clinical labs, NCEP standards for total and HDL-c are more stringent. The use of repeated measures has been previously shown to improve risk prediction by reducing regression dilution bias and providing more stable risk factor values [29]. Therefore, in patients with borderline risk (e.g. between 5-7.5 %), it may be prudent to perform repeat measurements (at a certain time apart, preferably triplicate measurements). A similar approach has been previously proposed in NCEP Adult Treatment Panel III guidelines and in a US Preventive Services Taskforce statement that recommends repeating the lipid profile to confirm abnormal values [http://www.uspreventiveservicestaskforce.org/uspstf08/lipid/lipidrs.htm [30],]. In addition, use of NCEP network laboratories may be prudent in certain situation such as of wide variability in the measurement values.

It should be emphasized that clinical judgement and discussion with the individual patient is important when deciding for optimal treatment approach based on calculated ten-year risk. This aspect has also been highlighted in the ten-year risk guidelines. Risk equations provide important guidance to the clinicians and the patients for such discussion. However since these equations represent mathematical functions/ best fit based on the results of prospective cohort studies, they do have inherent uncertainties and hence cannot supersede the judgement of a clinician. Our analysis does have important limitations. The distribution function of the variance of the continuous variables in actual clinical situations is not well described. We calculated the outermost boundaries of ten-year risk and the net uncertainty assuming uniform distribution and predictable direction in the variance of each variable. This therefore would result in greater net risk re-categorization than in clinical situations. However, we have provided a practical framework for estimating the impact of uncertainties in important clinical variables. Assessment of the whole spectra of intermediate effects due to bidirectional uncertainties in clinical variable measurements as well as the assessment of relative role of specific input variables on the risk re-categorization was not considered in the present study. The relative interaction of clinical input variable uncertainties and model inherent uncertainties (i.e., group risk prediction intervals) in individual risk predictions [19], was also beyond the scope of the present study. Such analysis would require much more sophisticated simulation and incorporating data from large clinical datasets.

Based on our previous publications [17, 18] and present work, we would suggest two additional features for the future iterations of the risk calculator. Firstly, it should allow the customizable input of uncertainty limits for relevant variables based on the local or reference laboratory standards. Further, it should estimate the upper and lower boundaries for ten-year ASCVD risk taking into account various uncertainties including that for individual datasets and model fits.

## Conclusions

Our intent in writing this manuscript is to raise awareness about certain aspects of the new pooled cohort equations for ten-year risk calculation that are not immediately apparent. We describe effects of the uncertainty in measurements of important variables for calculating ten-year risk that may have a significant impact in preventive approaches to ASCVD. Incorporating good clinical practices for the measurement of critical clinical variables and robust standardization of laboratory parameters to more stringent reference standards is extremely important for successful implementation of the new guidelines. Furthermore, ability to customize the risk calculator inputs to better represent unique clinical circumstances specific to individual needs would be highly desirable in the future versions of the risk calculator.

## Additional files

Additional file 1: Dataset: our detailed analysis dataset of NHANES data. (XLSX 319 kb) Additional file 2: Table S1. Analysis of the impact of input variable variations in categorizing subjects based on ten-year risk threshold of 7.5 % (with Hispanics). (DOCX 15 kb) Additional file 3: Table S2. Analysis of the impact of input variable variations in categorizing subjects based on ten-year risk threshold of 5 % (with Hispanics). (DOCX 14 kb)

## Abbreviations

AA: African-American
ACC/AHA: American College of Cardiology/American Heart Association
ARIC: Atherosclerosis risk in communities study
ASCVD: Atherosclerotic cardiovascular disease
BP: Blood pressure
c: Cholesterol
CARDIA: Coronary artery risk development in young adults
CHS: Cardiovascular health study
CLIA: Clinical laboratory improvement amendment
DM: Diabetes mellitus
FHS: Framingham heart study
HDL: High density lipoprotein
MESA: Multi-ethnic study of atherosclerosis
NCEP: National cholesterol education program
NHANES: National health and nutrition examination survey
REGARDS: Reasons for geographic and racial differences in stroke
