# Supplementary material for: Impact of clinical input variable uncertainties on ten-year atherosclerotic cardiovascular disease risk using new pooled cohort equations
Source: BMC Cardiovasc Disord. 2016 Aug 31;16(1):165. doi: 10.1186/s12872-016-0352-x (PMC5007709; doi:10.1186/s12872-016-0352-x)
Supplement: Additional file 2: Table S1. — Analysis of the impact of input variable variations in categorizing subjects based on ten-year risk threshold of 7.5 % (with Hispanics). (DOCX 15 kb) [file 12872_2016_352_MOESM2_ESM.docx]

**Table S1**. Analysis of the impact of input variable variations in categorizing subjects based on ten-year risk threshold of 7.5% (with Hispanics)

| Patient groups | Base Calculated Ten Year Risk < 7.5% (% of total) | | | Base Calculated Ten Year Risk ≥ 7.5% (% of total) | | | Total Change of Risk Categorization  (% of total) |
| --- | --- | --- | --- | --- | --- | --- | --- |
|  | Base Calculated Risk <7.5% | No change of risk categorization (Maximal calculated risk <7.5%) | Change of risk categorization (Maximal calculated risk ≥7.5%) | Base Calculated Risk ≥7.5% | No change of risk categorization (Minimal calculated risk ≥7.5%) | Change of risk categorization (Minimal calculated Risk <7.5% |  |
| All(n= 2355) | 33.33 | 20.98*** | 12.35 | 66.67 | 54.82*** | 11.85 | 24.20 |
| Non-DM(n=1601) | 43.22 | 28.04*** | 15.18 | 56.78 | 45.10*** | 11.68 | 26.86 |
| AA(n=426) | 36.15 | 20.42*** | 15.73 | 63.85 | 52.11*** | 11.74 | 27.46 |
| AA Male(n=196) | 14.29 | 6.63* | 7.66 | 85.71 | 78.57 | 7.14 | 14.80 |
| AA Female(n=230) | 54.78 | 32.17*** | 22.61 | 45.22 | 29.57*** | 15.65 | 38.26 |
| White(n=1175) | 45.79 | 30.81*** | 14.98 | 54.21 | 42.55*** | 11.66 | 26.64 |
| White Male(n=532) | 33.46 | 18.98*** | 14.48 | 66.54 | 53.20*** | 13.34 | 27.82 |
| White Female(n=643) | 55.99 | 40.59*** | 15.4 | 44.01 | 33.75*** | 10.26 | 25.66 |
| DM(n=754) | 12.33 | 5.97*** | 6.36 | 87.67 | 75.46*** | 12.21 | 18.57 |
| AA(n=255) | 5.88 | 2.35 | 5.53 | 94.12 | 84.71*** | 9.41 | 12.94 |
| AA Male(n=107) | 0.00 | 0.00 | 0 | 100.00 | 98.13 | 1.87 | 1.87 |
| AA Female(n=148) | 10.14 | 4.05 | 6.09 | 89.86 | 75.00** | 14.86 | 20.95 |
| White(n=499) | 15.63 | 7.82*** | 7.81 | 84.37 | 70.74*** | 13.63 | 21.44 |
| White Male(n=220) | 8.64 | 3.18* | 5.46 | 91.36 | 82.73* | 8.63 | 14.09 |
| White Female(n=279) | 21.15 | 11.47** | 9.68 | 78.85 | 61.29*** | 17.56 | 27.24 |

Values are % or n. Base calculated: predicted ten-year risk using the raw NHANES data; Minimal Risk: minimum predicted ten-year risk computed by the calculator assuming a variation in age of 0 – 1 year, and ± 10% variation in total-cholesterol (c), HDL-c, and systolic blood pressure (BP); Maximal Risk: maximum predicted ten-year risk computed by the calculator assuming a variation in age of 0 – 1 year, and ± 10% variation in total-cholesterol (c), HDL-c, and systolic blood pressure (BP); Comparisons between Base versus Max/Min Risk were performed using Fisher’s Exact Test; * for P < 0.05, ** for P < 0.01, and *** for P < 0.001.
