# Supplementary material for: Impact of clinical input variable uncertainties on ten-year atherosclerotic cardiovascular disease risk using new pooled cohort equations
Source: BMC Cardiovasc Disord. 2016 Aug 31;16(1):165. doi: 10.1186/s12872-016-0352-x (PMC5007709; doi:10.1186/s12872-016-0352-x)
Supplement: Additional file 3: Table S2. — Analysis of the impact of input variable variations in categorizing subjects based on ten-year risk threshold of 5 % (with Hispanics). (DOCX 14 kb) [file 12872_2016_352_MOESM3_ESM.docx]

**Table S2**. Analysis of the impact of input variable variations in categorizing subjects based on ten-year risk threshold of 5% (with Hispanics)

| Patient groups | Base Calculated Ten Year Risk < 5% (% of total) | | | Base Calculated Ten Year Risk ≥ 5% (% of total) | | | Total Change of Risk Categorization  (% of total) |
| --- | --- | --- | --- | --- | --- | --- | --- |
|  | Base Calculated Risk <5% | No change of risk categorization (Maximal calculated risk <5%) | Change of risk categorization (Maximal calculated risk ≥5%) | Base Calculated Risk ≥5% | No change of risk categorization (Minimal calculated risk ≥5%) | Change of risk categorization (Minimal calculated Risk <5% |  |
| Non-DM(n=1601) | 30.17 | 17.24*** | 12.93 | 69.83 | 58.34*** | 11.49 | 24.42 |
| AA(n=426) | 23.47 | 10.80*** | 12.67 | 76.53 | 65.49*** | 11.04 | 23.71 |
| AA Male(n=196) | 4.08 | 1.53 | 2.55 | 95.92 | 90.82 | 5.1 | 7.65 |
| AA Female(n=230) | 40.00 | 18.70*** | 21.3 | 60.00 | 43.91*** | 16.09 | 37.39 |
| White(n=1175) | 32.60 | 19.57*** | 13.03 | 67.40 | 55.74*** | 11.66 | 24.68 |
| White Male(n=532) | 22.18 | 11.09*** | 11.09 | 77.82 | 67.67*** | 10.15 | 21.24 |
| White Female(n=643) | 41.21 | 26.59*** | 14.62 | 58.79 | 45.88*** | 12.91 | 27.53 |

Values are % or n. Base calculated: predicted ten-year risk using the raw NHANES data; Minimal Risk: minimum predicted ten-year risk computed by the calculator assuming a variation in age of 0 – 1 year, and ± 10% variation in total-cholesterol (c), HDL-c, and systolic blood pressure (BP); Maximal Risk: maximum predicted ten-year risk computed by the calculator assuming a variation in age of 0 – 1 year, and ± 10% variation in total-cholesterol (c), HDL-c, and systolic blood pressure (BP); Comparisons between Base versus Max/Min Risk were performed using Fisher’s Exact Test; * for P < 0.05, ** for P < 0.01, and *** for P < 0.001.
